# *Lactobacillus reuteri*‐mediated dietary xylooligosaccharides enhance jejunal cell survival via suppression of oxygen‐dependent apoptotic processes in a pig model

**DOI:** 10.1002/imt2.70080

**Published:** 2025-09-13

**Authors:** Fuli Deng, Chang Yin, Chengzeng Luo, Ye Xu, Yuxia Chen, Ruqing Zhong, Shanlong Tang, Hongfu Zhang, Liang Chen

**Affiliations:** ^1^ State Key Laboratory of Animal Nutrition and Feeding, Key Laboratory of Animal Nutrition and Feed Science of the Ministry of Agriculture and Rural Affairs, Institute of Animal Science Chinese Academy of Agricultural Sciences Beijing China

## Abstract

Interactions between functional oligosaccharides and small intestinal cells are increasingly recognized as critical for maintaining intestinal health. Using xylooligosaccharides (XOS) as a model, we demonstrate that XOS promote growth in piglets primarily by enhancing nutrient transport and increasing villus height in the jejunum. These effects are mediated by XOS‐driven modulation of the intestinal microbiota, particularly the enrichment of *Lactobacillus reuteri* (*L. reuteri*) as a keystone species. *L. reuteri* reprograms epithelial energy metabolism toward reduced oxygen dependence, thereby inhibiting epithelial apoptosis and prolonging enterocyte survival. Notably, this protective effect is closely associated with elevated levels of the bile acid glycochenodeoxycholic acid (GCDCA), implicating a bile acid‐dependent mechanism. Together, these findings reveal a microbiota‐ and metabolite‐mediated pathway through which XOS regulate epithelial homeostasis and intestinal health.

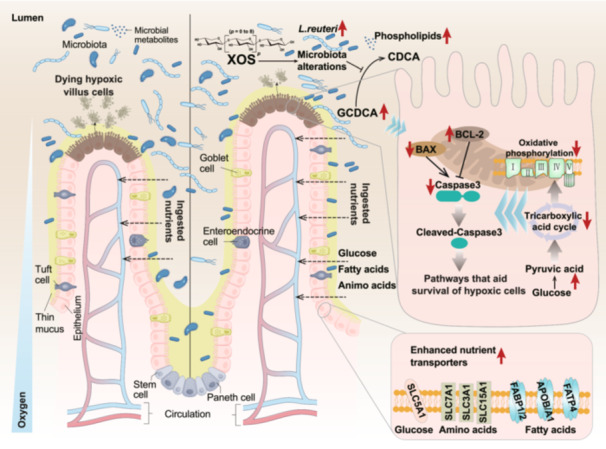

To the Editor,

The balance of gut microbiota critically influences mammalian growth and development [1, 2]. Functional oligosaccharides (FOS), such as xylooligosaccharides (XOS) composed of xylose monomers linked by β‐(1,4)‐glycosidic bonds, resist hydrolysis by host digestive enzymes and reach the distal colon for fermentation by probiotic strains including *Lactobacillus* and *Bifidobacterium* [3, 4, 5]. Although traditionally regarded as prebiotics, emerging evidence suggests that specific oligosaccharides can act directly on intestinal epithelial cells (IECs), strengthening barrier function and modulating immune responses [6, 7].

Here, we show that dietary XOS improved growth performance in young animals by promoting jejunal villus height and enhancing small intestinal health. These benefits were strongly associated with an optimized jejunal microbiota, most notably expansion of the *Lactobacillus* genus. While the gut microbiota is well known to ferment indigestible substrates in the hindgut to produce short‐chain fatty acids, modulate immunity, and maintain gut homeostasis [8], it also contributes to nutrient sensing and digestion in the small intestine, thereby influencing host energy balance [2, 9, 10].

Our results reveal that XOS supplementation optimizes small intestinal microbiota composition, including the proliferation of *Lactobacillus*, which in turn reduces epithelial apoptosis. This protection is closely associated with reduced oxygen dependence in IECs and alterations in luminal bile acid composition. Collectively, these findings uncover a mechanistic pathway through which dietary oligosaccharides regulate gut microbiota and promote epithelial cytoprotection.

## XOS improve growth performance by enhancing small intestinal morphology and barrier function

**FIGURE 1:**
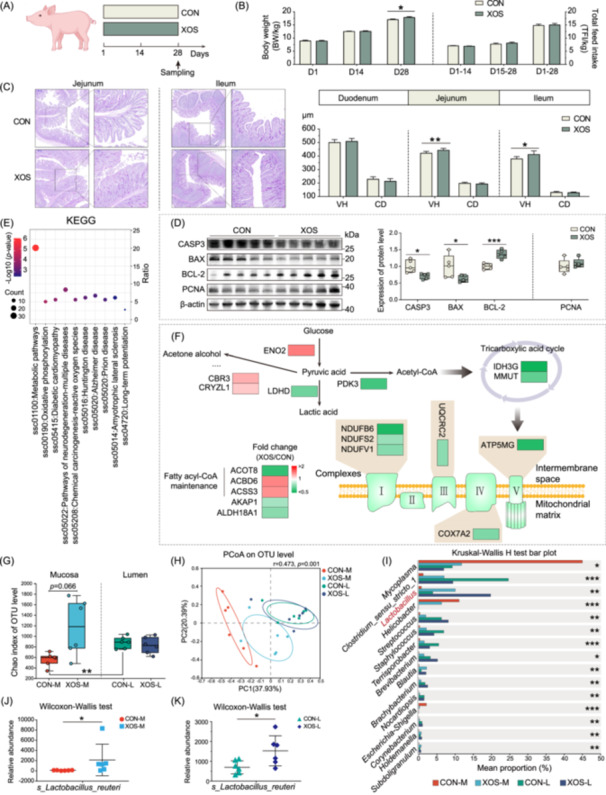
Dietary xylooligosaccharides (XOS) improve epithelial cell survival and growth performance in piglets through reduced apoptosis and microbiota remodeling. (A) Schematic diagram illustrating the experimental design: weaned piglets of similar body weight were randomly assigned to two groups and fed experimental diets for 28 days. CON: basal diet; XOS: basal diet supplemented with 500 mg/kg XOS. (B) Growth performance of piglets after XOS supplementation. (C) Quantitative indicators and representative images of small intestinal morphology. (D) Protein levels of cell apoptosis‐ and proliferation‐related genes in the jejunal mucosa of piglets after XOS supplementation. Values are mean ± SD or minimum to maximum with all points shown. A two‐tailed Student's *t*‐test was used for statistical analysis with asterisks denoting significant differences (*p < 0.05, **p < 0.01, and ***p < 0.001). Jejunal mucosal proteomics links XOS‐induced apoptosis suppression to metabolic reprogramming. (E) KEGG pathway analysis of differentially accumulated proteins. (F) Effects of XOS supplementation on proteins related to glucose glycolysis, the tricarboxylic acid cycle, oxidative phosphorylation, and fatty acid metabolism. Red indicates upregulated proteins; green indicates downregulated proteins. XOS optimize microbial composition and enhance the *Lactobacillus* abundance in the jejunal lumen and mucosa of piglets. (G) α‐diversity measured by Chao index at OTU level. (H) PCoA of the jejunal luminal and mucosal microbiome based on Bray–Curtis distance metrics at OTU level. (I) Nonparametric comparisons of microbial composition at the genus level. Wilcoxon–Wallis test for *Lactobacillus reuteri* (*L. reuteri*) abundance in the jejunal mucosa (J) and lumen (K). Nonparametric tests were used for α‐diversity, while Mann–Whitney *U* tests were used for microbial composition. Asterisks denote significant differences (*p < 0.05, **p < 0.01, and ***p < 0.001).

FOS supplementation has been shown to promote growth performance in livestock. To determine whether XOS exert similar effects, we assessed the impact of dietary XOS (Figure S1) on body weight and small intestinal morphology in piglets. As outlined in the experimental design (Figure 1A), piglets supplemented with XOS exhibited significantly increased body weight after 28 days (Figure 1B), along with a marked increase in villus length in both the jejunum and ileum (Figure 1C).

Given the critical role of epithelial barrier function in intestinal health, we examined tight junction markers in the jejunum. XOS significantly increased *ZO‐1* mRNA levels (Figure S2A) and showed a trend toward elevated protein expression of Claudin‐1 and ZO‐1 (Figure S2B). These results suggest that XOS promote growth by improving intestinal morphology and strengthening epithelial barrier integrity, consistent with previous findings in piglets and broilers [11, 12, 13].

## XOS inhibit epithelial cell apoptosis associated with alterations in energy metabolism in the jejunum

The small intestinal epithelium undergoes rapid turnover, renewing approximately every 3 days [14]. Villus length is determined by a balance between epithelial cell proliferation and apoptosis. In our study, XOS supplementation did not affect the expression of proliferation‐related genes, but significantly increased the antiapoptotic marker BCL‐2 while reducing proapoptotic CASP3 and BAX at both mRNA and protein levels (Figure 1D and Figure S2C). These data suggest XOS extend jejunal villus length primarily through inhibiting epithelial cell apoptosis.

To elucidate the underlying mechanisms, we profiled the jejunal mucosal proteome and identified 188 differentially accumulated proteins (DAPs), with 101 upregulated and 87 downregulated in XOS‐treated piglets (Figure S3A). Gene expression analysis of representative DAPs validated the proteomics results (Figure S4). Pathway enrichment analysis revealed that the altered proteins were predominantly involved in energy metabolism pathways (Figure 1E and Figure S3B−E).

We next focused on the function of energy metabolism‐related proteins in response to XOS treatment (Figure 1F). XOS supplementation increased levels of enzymes involved in glucose and pyruvate metabolism (ENO2 for glucose‐to‐pyruvate conversion; CBR3 and CRYZL1 for pyruvate‐to‐acetone conversion) and reduced enzymes catalyzing pyruvate conversion (LDHD for pyruvate‐to‐lactate; PDK3 for pyruvate‐to‐acetyl‐CoA). The abundance of enzymes in the tricarboxylic acid cycle (IDH3G, MMUT), mitochondrial electron transport chain complexes (Complex I: NDUFB6, NDUFS2, NDUFV1; Complex III: UQCRC2; Complex IV: COX7A2; Complex V: ATP5MG), and fatty acid metabolic enzymes (AKAP1, ALDH18A1, ACOT8) were also decreased. Collectively, these findings indicate that XOS suppress oxidative phosphorylation and the tricarboxylic acid cycle, thereby reducing cellular oxygen demand.

As oxygen availability is a critical determinant of epithelial cell survival and villus growth [15], the observed metabolic reprogramming likely contributes to reduced epithelial apoptosis and enhanced villus architecture under hypoxic stress.

## XOS enhance the expression of nutrient transporter and cytoskeleton‐related proteins

In addition to metabolic regulation, XOS treatment significantly altered cytoskeleton‐related proteins. Pathway analysis showed that DAPs were also enriched in cytoskeleton remodeling processes (Figure 1E and Figure S3B−E). Expression of proteins promoting microfilament assembly (PFDN6, XPO6, ANLN, EPB41L2, GMFB, FAM49A) was elevated, whereas MICAL1, a microfilament depolymerizing factor, was downregulated (Figure S5A,B). Moreover, proteins associated with myosin and villin (TPM1, TPM3, TPM4, MYH2, VBP1, and DPCD) were also increased (Figure S5C), collectively indicating active cytoskeletal remodeling in the jejunal mucosa following XOS treatment (Figure S5D).

These structural changes were accompanied by enhanced expression of nutrient transporters. The glucose transporter SLC5A10 and the starch‐derived metabolite transporter SI were markedly upregulated in XOS‐treated piglets (Figure S5E). In addition, qRT‐PCR confirmed increased expression of the principal glucose transporter *SLC5A1* (*SGLT1*; Figure S5F). Furthermore, the mRNA levels of lipid transporters (*FATP4*, *CD36*, *FABP2*, *FABP1*, *APOA1*, *APOB*) and several amino acid transporters (*SLC7A1*, *SLC3A1* and *SLC15A1)* were also upregulated in the jejunum (Figure S5G,H).

Together, these findings indicate that dietary XOS enhance nutrient absorption by coordinately regulating cytoskeletal remodeling and nutrient transporter expression.

## XOS optimize jejunal microbial composition and enhance the *Lactobacillus reuteri* abundance

Xylobiose or xylotriose, the major components of XOS, may directly interact with intestinal epithelial cells to modulate cellular functions [16]. To evaluate whether they protect epithelial cells against hypoxic stress, we assessed apoptosis in IPEC‐J2 cells. Dose optimization first established the CoCl_2_ concentration that reduced cell viability to ~80%, as determined by CCK‐8 assay (Figure S6A,B). This concentration significantly upregulated the apoptotic markers CASP3 and BAX, but not BCL‐2 (Figure S6E). At non‐cytotoxic concentrations (Figure S6C,D), neither xylobiose nor xylotriose attenuated the hypoxia‐induced upregulation of CASP3 and BAX when applied alone (Figure S6F), indicating limited protective effects against hypoxia‐induced epithelial apoptosis.

Given the reliance of XOS on microbial metabolism and the central role of host–microbe interactions in dietary responses and nutrient sensing [10], we next investigated the effect of XOS supplementation on jejunal microbiota. Measures of α‐diversity (Chao/Sobs indices) and PCoA revealed clear differences between mucosal and luminal microbial communities. Specifically, XOS induced significant shifts in α‐diversity within the jejunal mucosa but not in the luminal digesta (Figure 1G and Figure S7A). PCoA (Bray–Curtis) confirmed marked restructuring of mucosal microbiota (Figure 1H and Figure S7B), while luminal microbiota remained relatively stable at the OTU level (Figure 1H and Figure S7C).

At the phylum level, Firmicutes dominated both mucosa (~50%) and lumen (>68%), followed by Proteobacteria/Campilobacterota in mucosa and Proteobacteria/Actinobacteria in lumen (Figure S7D). At the genus level, XOS significantly increased beneficial *Lactobacillus*, *Streptococcus*, and *Brevibacterium* in both compartments (Figure 1I and Figure S7E−G). In contrast, potentially pathogenic *Terrisporobacter* decreased in the lumen but increased in the mucosa; *Escherichia‐Shigella* decreased in the mucosa, suggesting inhibited colonization (Figure 1I and Figure S7E−G). Species‐level profiling identified *Lactobacillus reuteri* (*L. reuteri*) as the principal driver of *Lactobacillus* expansion (Figure 1J,K, and Table S1), with additional enrichment of *Bifidobacterium* in the luminal chyme following XOS supplementation (Figure S7G).

Correlation network analysis demonstrated that XOS increased both the connectivity and strength of microbial associations in the jejunal mucosa and lumen. Notably, *Lactobacillus* emerged as a central hub species within these networks (Figure S8). Collectively, these findings suggest that XOS reshape the jejunal microbiota, prominently enriching *L. reuteri* and thereby optimizing the microbial ecosystem of the proximal intestine.

### *L. reuteri* supplementation reverses *E. coli*‐induced villus injury in piglets

**FIGURE 2:**
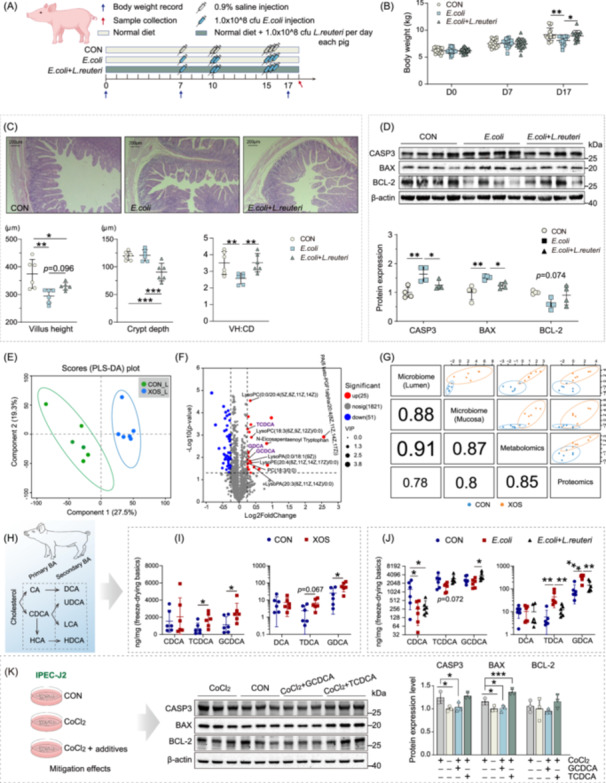
*Lactobacillus reuteri* (*L. reuteri*) ‐mediated metabolism of dietary xylooligosaccharides (XOS) elevates glycochenodeoxycholic acid (GCDCA), enhancing hypoxic tolerance in jejunal epithelium. (A) Schematic diagram of the experimental design for *L. reuteri* supplementation in *E. coli*‐challenged piglets. CON group: basal diet; *E. coli* group: basal diet plus multiple *E. coli* injections; *E. coli* + *L. reuteri* group: basal diet supplemented with 1.0 × 10^8^ CFU *L. reuteri* per pig per day plus multiple *E. coli* injections. (B) Body weight of piglets after *E. coli* injection with or without *L. reuteri* supplementation. (C) Quantitative indicators and representative images of jejunum morphology. (D) Protein levels of apoptosis‐related genes in the jejunal mucosa of piglets after *E. coli* challenge with or without *L. reuteri* supplementation. Values represent mean ± SD. Metabolomic and multi‐omics correlation analyses identify bile acids and lysophospholipids as key metabolites. Metabolomic profiling of jejunal mucosa from XOS‐treated pigs: (E) PLS‐DA score plot showing separation between CON and XOS groups; (F) Volcano plot highlighting differentially abundant metabolites. (G) Global correlation structure among proteome, microbiome (jejunal lumen and mucosa) and metabolome (jejunal digesta) by DIABLO (Data Integration Analysis for Biomarker discovery using a Latent component method for Omics). (H) Bile acid composition in the pig model. Targeted metabolomics confirming bile acid contents of jejunal digesta in (I) XOS‐fed and (J) *E. coli* + *L. reuteri* groups. (K) Relative expression of apoptosis‐related proteins in IPEC‐J2 cells under CoCl₂‐induced hypoxia (300 μM) with or without GCDCA/TCDCA supplementation (0.1 μM). Values are expressed as mean ± SD. The two‐tailed Student's *t*‐test and one‐way ANOVA were used for two groups and three groups statistical analysis; After a significant ANOVA result, post hoc comparisons were performed using Duncan's multiple range test, and asterisks denote significant differences (*p < 0.05, **p < 0.01, and ***p < 0.001).

To assess whether XOS‐induced *L. reuteri* enrichment contributes to intestinal epithelial cell survival, we examined the effects of *L. reuteri* supplementation in an *E. coli*‐induced villus injury model (Figure 2A). Repeated *E. coli* injections caused weight loss in piglets, which was mitigated by *L. reuteri* treatment (Figure 2B). Histological analysis revealed reduced jejunal villus height following *E. coli* challenge, whereas *L. reuteri* tended to restore villus height (*p* = 0.096) (Figure 2C). Although crypt depth was unaffected by *E. coli* alone, *L. reuteri* significantly reduced crypt depth in challenged piglets, thereby improving the villus‐to‐crypt (V/C) ratio and restoring it to control levels (Figure 2C).

To investigate mechanisms underlying these morphological improvements, we assessed apoptosis and proliferation markers. At the mRNA level, only proapoptotic marker *CASP3* was significantly altered, with *L. reuteri* reversing its upregulation induced by *E. coli* (Figure S9A). Protein analysis confirmed reduced expression of CASP3 and BAX following *L. reuteri* treatment (Figure 2D), whereas antiapoptotic BCL‐2 showed a trend of increase compared with the *E. coli* group (*p* = 0.074, Figure 2D). The proliferation marker PCNA was not significantly affected, with only a modest reduction in the *E. coli* + *L. reuteri* group (*p* = 0.074) (Figure S9B). These results indicate that *L. reuteri* attenuates villus injury primarily by suppressing apoptosis rather than stimulating proliferation. Notably, previous studies have shown that individual microbial strains, such as *L. rhamnosus* and *L. reuteri*, can promote intestinal proliferation via NADPH oxidase 1‐dependent ROS generation [17], or dietary fructose production [18], respectively.

*L. reuteri* also normalized expression of several energy metabolism‐related genes upregulated by *E. coli* challenge (*PDK3*, *ATP5MG*, *IDH3G*, *MMUT*, *NDUFB6*, *NDUFV1*), restoring them to control levels (Figure S9C). Cytoskeleton‐related genes, *ANLN* and *TPM4*, showed modest increases in the *E. coli* + *L. reuteri* group (0.05 < *p* < 0.10, Figure S9D). Among nutrient transporters, *SLC7A1*, *SLC7A9*, and *CD36* were upregulated, while no significant differences were observed for other glucose, amino acid, or fatty acid transporters (Figure S9E−G).

These data suggest that the protective effect of *L. reuteri* against *E. coli*‐induced villus injury is mediated largely through modulation of apoptosis and energy metabolism, with limited contributions from cytoskeletal remodeling and transporter regulation.

## Metabolomics and multi‐omics correlation analysis identify potential metabolites mediating *L. reuteri*‐dependent protection

We hypothesized that the antiapoptotic effects of XOS‐enriched microbiota, dominated by *L. reuteri*, are mediated through microbial metabolites interacting with intestinal epithelium. Untargeted metabolomics of jejunal chyme from XOS‐fed pigs identified 25 upregulated metabolites, among which bile acids and lysophospholipids emerged as potential mediators (Figure 2E,F, and Figure S10). Notably, lysophospholipids were largely absent from *L. reuteri* cultures and supernatants, except for elevated PA (16:0/18:2(9Z,12Z)) (Figure S11), indicating that relevant lysophospholipids are not directly produced by *L. reuteri*, but arise through cooperative metabolism within the gut microbial community.

To integrate these findings, we performed multi‐omics correlation analysis of jejunal microbiota (lumen/mucosa), chyme metabolome, and mucosal proteome in XOS‐treated piglets. The chyme metabolome showed a strong correlation with luminal microbiota (*r* = 0.91, Figure 2G), while the mucosal proteome was more tightly linked to the metabolome than to microbiota (Figure 2G), supporting the role of metabolites as regulators of epithelial metabolism and apoptosis. Among the upregulated metabolites (Figure S12A), bile acids including glyco/tauro‐chenodexycholic acids (G/TCDCA) and glycodeoxycholic acid (GDCA) positively correlated with luminal *Peptococcus* and *Lactobacillus*, and with several mucosal taxa, including *Clostridium sensu stricto 1/6*, *Eubacterium xylanophilum*, *Collinsella*, *Terrisporobacter*, *Catenisphaera*, *E. nodatum*, *Butyricicoccus*, *Enterorhabdus*, and *Eggerthellaceae*. Lysophospholipid‐related LysoPC species correlated with multiple mucosa‐resident bacteria. Importantly, both bile acids and lysophospholipids showed positive correlations with jejunal villus height (Figure S12B).

Targeted metabolomics further quantified bile acids in jejunal chyme from XOS‐fed and *E. coli* + *L. reuteri* groups. XOS significantly increased GCDCA/TCDCA and modestly elevated GDCA (Figure 2H,I). Furthermore, *E. coli* challenge reduced chenodeoxycholic acid (CDCA) and GCDCA/TCDCA, while *L. reuteri* restored GCDCA/TCDCA to control levels. Conversely, T/GDCA increased following *E. coli* challenge but decreased after *L. reuteri* treatment (Figure 2H,J). Additionally, bile acid profiling showed reduced cholic acid (CA, *p* = 0.070) and hyocholic acid (HCA) in *E. coli*‐challenged pigs, whereas *L. reuteri* suppressed *E. coli*‐induced elevation of tauro‐CA (TCA) and increased tauro‐LCA (TLCA) (Figure S13).

## Low‐dose GCDCA attenuates hypoxia‐induced apoptosis of IPEC‐J2 cells in vitro

Bile acids are key regulators of intestinal homeostasis [19], and CDCA has been reported to protect against LPS‐induced epithelial damage [20]. We thus hypothesized that its conjugated form, GCDCA, might ameliorate hypoxia‐induced apoptosis. To test this, we applied a CoCl_2_‐induced hypoxia model in IPEC‐J2 cells to screen candidate antiapoptotic metabolites.

Dose–response assays defined cytotoxicity thresholds of GCDCA and TCDCA in IPEC‐J2 cells. Cell viability remained unaffected at concentrations up to 50 μM GCDCA and 100 μM TCDCA after a 24‐h incubation (Figure S14A−C). At high doses (50 μM), both GCDCA and TCDCA synergized with CoCl_2_ to induce vacuolation‐associated cell death, while concentrations as low as 10 μM caused cell detachment. In contrast, lower concentrations (≤1 μM) did not cause morphological alterations compared to CoCl_2_ alone (Figure S14D). Strikingly, 0.1 μM GCDCA significantly attenuated CoCl_2_‐induced upregulation of CASP3 and BAX, whereas TCDCA conferred no protection (Figure 2K). These results demonstrate that low‐dose GCDCA exerts a direct antiapoptotic effect under hypoxia conditions.

Our study reveals a previously unrecognized function of non‐digestible XOS in the proximal intestine: attenuation of enterocyte apoptosis through enrichment of *L. reuteri* in the jejunum. XOS supplementation improved growth performance by increasing jejunal villus height and enhancing nutrient transport in weaned piglets. Mechanistically, *L. reuteri* enrichment reshaped epithelial energy metabolism toward reduced oxygen dependence, thereby promoting epithelial cell survival under hypoxia conditions. A critical outcome of this process was the accumulation of GCDCA, identified as a key metabolite mediating hypoxia resistance.

These findings challenge the prevailing view that oligosaccharides transit the small intestine passively, instead revealing an active role for XOS in modulating host‐microbiota interactions and highlighting the contribution of jejunal microbiota to epithelial cytoprotection and intestinal homeostasis.

All the materials and methods are described in the Supporting Information. The abbreviations correspondence table is detailed in Table S5.

## AUTHOR CONTRIBUTIONS

**Fuli Deng**: Software; data curation; investigation; validation; formal analysis; methodology; visualization. **Chang Yin**: Data curation; investigation; validation; formal analysis; visualization. **Chengzeng Luo**: Data curation; investigation; methodology. **Ye Xu**: Data curation; investigation. **Yuxia Chen**: Data curation; methodology. **Ruqing Zhong**: Resources. **Shanlong Tang**: Conceptualization; supervision; visualization; writing—original draft; project administration. **Hongfu Zhang**: Writing—review and editing; conceptualization; methodology; supervision. **Liang Chen**: Conceptualization; funding acquisition; writing—review and editing; project administration; supervision.

## CONFLICT OF INTEREST STATEMENT

The authors declare no conflicts of interest.

## ETHICS STATEMENT

The ethics applications (IAS2019‐34 and IAS2021‐35) were approved by the Experimental Animal Welfare and Ethical Committee of the Institute of Animal Science of Chinese Academy of Agricultural Sciences (IAS‐CAAS).

## Supporting information


**Figure S1:** The components of XOS and their corresponding retention times.
**Figure S2:** Gene expression related to intestinal tight junction, cell apoptosis or proliferation after XOS supplement.
**Figure S3:** Enrichment analysis of RNA sequencing.
**Figure S4:** Validation of proteomics analysis results.
**Figure S5:** The expression of nutrient transporter genes and cytoskeleton‐related proteins from proteomic or qRT‐PCR data.
**Figure S6:** The hypoxia‐induced apoptosis model and effects of xylobiose or xylotriose on attenuating hypoxia‐induced apoptosis of IPEC‐J2 cells *in vitro*.
**Figure S7:** The changes of microbial composition.
**Figure S8:** A network for correlation analysis in the relative abundances of microbiota genera.
**Figure S9:** Protein and gene expression in jejunal mucosa after *E. coli* injection with or without *L. reuteri* supplement.
**Figure S10:** Differential metabolite profile for jejunal digesta (CON vs XOS).
**Figure S11:** Metabolomics of bacteria.
**Figure S12:** Metabolomic data and multi‐omics correlation analysis.
**Figure S13:** Bile acid contents.
**Figure S14:** Cytotoxicity and tolerance experiments of exogenous additives.

**Table S1:** XOS enriched the composition proportions of different *Lactobacillus* species in jejunal mucosa and lumen (*n* = 6).
**Table S2:** Composition and nutrient levels of the corn‐soybean basal diet (air‐dry basis).
**Table S3:** The information of specific primer sequences used in qRT‐PCR.
**Table S4:** Primary antibody information.
**Table S5:** Abbreviation information.

## Data Availability

The raw sequence data of jejunal mucosa or lumen microbiota during this study have been deposited in the NCBI Sequence Read Archive (SRA; PRJNA1266879, https://www.ncbi.nlm.nih.gov/bioproject/PRJNA1266879). The proteomics and metabolomics data reported in this paper have been deposited in the OMIX, China National Center for Bioinformation/Beijing Institute of Genomics, Chinese Academy of Sciences (PRJCA040592 (OMIX010295, https://ngdc.cncb.ac.cn/omix/release/OMIX010295) for metabolome and PRJCA041297 (OMIX010468, https://ngdc.cncb.ac.cn/omix/release/OMIX010468) for proteome). The data and scripts for analysis and visualization are saved in GitHub https://github.com/Tang-shanlong93/Tang2025iMeta. Supplementary materials (methods, figures, tables, graphical abstract, slides, videos, Chinese translated version, and update materials) may be found in the online DOI or iMeta Science http://www.imeta.science/.
