# Supplementary material for: Lactobacillus reuteri‐mediated dietary xylooligosaccharides enhance jejunal cell survival via suppression of oxygen‐dependent apoptotic processes in a pig model
Source: Imeta. 2025 Sep 13;4(5):e70080. doi: 10.1002/imt2.70080 (PMC12527980; doi:10.1002/imt2.70080)
Supplement: Supplementary file 1 — Figure S1: The components of XOS and their corresponding retention times. Figure S2: Gene expression related to intestinal tight junction, cell apoptosis or proliferation after XOS supplement. Figure S3: Enrichment analysis of RNA sequencing. Figure S4: Validation of proteomics analysis results. Figure S5: The expression of nutrient transporter genes and cytoskeleton‐related proteins from proteomic or qRT‐PCR data. Figure S6: The hypoxia‐induced apoptosis model and effects of xylobiose or xylotriose on attenuating hypoxia‐induced apoptosis of IPEC‐J2 cells in vitro. Figure S7: The changes of microbial composition. Figure S8: A network for correlation analysis in the relative abundances of microbiota genera. Figure S9: Protein and gene expression in jejunal mucosa after E. coli injection with or without L. reuteri supplement. Figure S10: Differential metabolite profile for jejunal digesta (CON vs XOS). Figure S11: Metabolomics of bacteria. Figure S12: Metabolomic data and multi‐omics correlation analysis. Figure S13: Bile acid contents. Figure S14: Cytotoxicity and tolerance experiments of exogenous additives. [file IMT2-4-e70080-s001.docx]

**Supporting information to**

***Lactobacillus reuteri*-mediated dietary xylooligosaccharides enhance jejunal cell survival via suppression of oxygen-dependent apoptotic processes in a pig model**

Fuli Deng^#^, Chang Yin^#^, Chengzeng Luo^#^, Ye Xu, Yuxia Chen, Ruqing Zhong, Shanlong Tang*, Hongfu Zhang*, Liang Chen*

State Key Laboratory of Animal Nutrition and Feeding, Key Laboratory of Animal Nutrition and Feed Science of the Ministry of Agriculture and Rural Aﬀairs, Institute of Animal Science, Chinese Academy of Agricultural Sciences, Beijing, China

^#^These authors contributed equally: Fuli Deng, Chang Yin, Chengzeng Luo

*Correspondence: [tangshanlong01@126.com](mailto:tangshanlong01@126.com) (Shanlong Tang), [zhanghongfu@caas.cn](mailto:zhanghongfu@caas.cn) (Hongfu Zhang), [chenliang01@caas.cn](mailto:chenliang01@caas.cn) (Liang Chen)

**METHODS**

**Animal experiments**

For the XOS treatment experiment, a total of 120 healthy, weaned piglets (Duroc × Landrace × Yorkshire, DLY; 28 days old) with a similar body weight (8.88 ± 0.15 kg) were randomly distributed into 8 pens, with 15 pigs per pen. The piglets were divided into two treatment groups, each consisting of 4 pens (n = 60). The control (CON) group received a basal diet without antibiotics or prebiotics, while the XOS treatment group was fed a basal diet supplemented with 500 mg/kg of XOS. The XOS, derived from corncob and provided by Longlive Bio-Technology Co., Ltd. (Shandong, China), has a purity of 95%, a degree of polymerization ranging from 2 to 7 (Figure S1). The experiment lasted for a period of 28 days (Figure 1A).

In the *L. reuteri* supplementation experiment, 54 healthy, weaned DLY piglets (21 days old) with a similar body weight of 6.20 ± 0.51 kg were randomly assigned to 9 pens, with 6 pigs per pen. The piglets were divided into three treatment groups: the CON group and the *E. coli* group, both receiving a basal diet without antibiotics or prebiotics, and the *E. coli* + *L. reuteri* group, which was fed a basal diet supplemented with 1.0 × 10^8^ CFU of *L. reuteri* per day each pig. On the 7th, 10th, and 15th days of the experiment, pigs in the *E. coli* and *E. coli* + *L. reuteri* group were injected with 1mL, 2mL, and 3mL of *E. coli* at a concentration of 1.0 × 10^8^ CFU/mL, respectively, while pigs in the CON group received an equivalent volume of 0.9% saline at the same time points. The *E. coli* and *L. reuteri* strains were sourced from our laboratory's self-screened pool. The formal trial phase lasted 17 days (Figure 2A).

**Animal management and sample collection**

All pigs were kept in a controlled environment with an ambient temperature of 25−28°C and relative humidity ranging from 60% to 70%, with *ad libitum* access to both feed and water. The diet was formulated according to the National Research Council (NRC, 2012) guidelines, primarily consisting of ground corn-soybean basal meal, and was free of antibiotics (Table S2).

At the termination of each animal experiment, 6-8 pigs were randomly selected from each group and anesthetized via intravenous injection of sodium pentobarbital at a dose of 50 mg/kg body weight. Following exsanguination, the abdomen was opened, and the entire small intestine was immediately isolated and rinsed with phosphate-buffered saline (PBS). A 1.5 cm segment of the small intestine was then placed in 4% freshly prepared paraformaldehyde solution for staining. Jejunal mucosal samples were collected by scraping with a glass slide, followed by snap freezing in liquid nitrogen and storage at −80°C for subsequent analysis. Additionally, jejunal digesta were aseptically collected into 2-mL tubes, frozen in liquid nitrogen, and stored at −80°C for microbial 16S gene sequencing and metabolomic analysis.

**Cell culture and treatment**

The intestinal porcine epithelial cell (IPEC-J2), a non-transformed intestinal epithelial cell model derived from the jejunum of neonatal piglets, was utilized for the experiments. The IPEC-J2 (RRID: CVCL_2246) was a generous gift from Dr. Zhengqun Liu (Tianjin Academy of Agriculture Sciences). Cells were cultured in a 1:1 mixture of Dulbecco’s modified Eagle medium (DMEM) and Ham’s F-12 medium (Gibco, ThermoFisher, MA, USA) supplemented with 10% inactivated fetal bovine serum (FBS; Gibco), 1% penicillin-streptomycin (Gibco), and 5 ng/mL epidermal growth factor (EGF; Sigma-Aldrich, MO, USA). Cultures were incubated at 37℃ in a humidified environment with 5% CO_2_. Cells were passaged at 70-80% confluency using 0.25% trypsin-EDTA (Gibco), and cell density was quantified using an automated cell counter (Invitrogen, ThermoFisher).

To determine optimal treatment concentrations, IPEC-J2 cells were seeded in 96-well plates (1×10^4^ cells/per well) and allowed to adhere for 24 h. Cells were then exposed to gradient concentrations of cobalt chloride (CoCl_2_; Sigma-Aldrich), glycol/tauro-chenodeoxycholic acid sodium (G/TCDCA; Macklin, Shanghai, China), or xylobiose/xylotriose (Sigma-Aldrich) in DEPC-treated water for 24 h. After 24 hours of treatment, cell viability was assessed using the CCK-8 method. For the formal experiments, cells were pre-cultured in 6-well plates (2×10^5^ cells/per well) for 24 h. Hypoxic conditions were chemically induced by treating cells with 300 μM CoCl₂ for 12 h; Concurrently, G/TCDCA or xylobiose/xylotriose was administered. Control groups included untreated cells and CoCl_2_-only treated cells. Finally analyses included assessments of cell viability, protein expression, and other relevant assays.

**Cytotoxicity assay by CCK-8 method**

Cell viability was determined using the mitochondrial dehydrogenase activity-based CCK-8 assay kit (Solarbio), where viable cells reduce 2-(2-methoxy-4-nitrophenyl)-3-(4-nitrophenyl)-5-(2,4-disulfophenyl)-2H-tetrazolium (WST-8) to water-soluble orange-yellow formazan product. Post-treatment, cells were rinsed twice with PBS. Then, CCK-8 reagent was directly added to each well at a 10% (v/v) ratio and incubated in the dark for 3 hours to facilitate chromogenic development. Subsequently, the absorbance of the resulting solution was quantified at 450 nm using a microplate spectrophotometer (Spectramax M2, Molecular Devices, USA). All experiments were conducted twice, with results normalized as a percentage of the control group.

**Periodic Acid-Schiff (PAS) staining**

The PAS staining of the jejunal tissue was carried out following the method described by [1]. In brief, paraformaldehyde-fixed jejunal segments were first dehydrated through a graded series of increasing ethanol concentration, followed by cleaning with xylene. The samples were then embedded in paraffin, sectioned into approximately 5-μm-thick cross-sections, and stained with PAS solution. Photomicrographs were captured using a Leica DM2000 light microscope (Leica Microsystems, Germany).

**Hematoxylin and Eosin (H&E) staining**

The H&E staining was performed following the protocol described by [2]. Briefly, jejunal tissue blocks were fixed, dehydrated, and embedded in paraffin. The paraffin blocks were sectioned, mounted on slides, dried, and deparaffinized. The slides were then stained with hematoxylin and eosin, dehydrated, and mounted with a coverslip before being analyzed under a microscope.

**Gene expression detection by quantitative real-time PCR (qRT-PCR)**

Total RNA was extracted from jejunal mucosa using the Total RNA Rapid Extraction Kit (GeneBetter Biotech, Beijing, China) following the manufacturer's instructions. The RNA concentration of each sample was determined using a NanoDrop spectrophotometer (Nanodrop Technologies, DE, USA). To eliminate genomic DNA contamination, samples were treated with gDNA Eraser from the PrimeScript™ RT reagent kit (Takara, Shige, Japan) before reverse transcription. RNA was then reverse transcribed into cDNA using the same reagent kit. Quantitative real-time PCR (qRT-PCR) was performed using SYBR® Premix Ex Taq™ (Takara), in accordance with the manufacturer's protocol. The specificity of each primer set (Table S3) was confirmed by checking for a single peak in the melting curves following 40 PCR cycles. Relative expression level of targeted genes in different groups was normalized to *β-actin* and *GAPDH* mRNA levels and calculated using the 2^−△△Ct^ method.

**Protein expression detection by western blotting**

Total proteins from jejunal mucosa or IPEC-J2 cells were isolated using RIPA lysis buffer (Solarbio) supplemented with protease inhibitor cocktail (Roche, Basel, Switzerland) and phosphatase inhibitors (Solarbio). Protein concentration was determined using the BCA protein assay kit (Beyotime, Shanghai, China). Following protein denaturation by boiling, samples were separated by 10 % SDS-PAGE gel electrophoresis and transferred to PVDF membranes via the wet transfer method. Membranes were blocked with 5 % skim milk at room temperature and incubated overnight at 4℃ with primary antibodies (Table S4). The following day, membranes were washed and then incubated with the HRP-labeled secondary antibody for 40 minutes at room temperature. Finally, protein bands were visualized using SuperSignal® West Femto Maximum Sensitivity Substrate (Thermo Fisher) and imaged with a gel imaging system (Tanon Science & Technology, Shanghai, China). Band density was quantified using Image J software and normalized to the reference protein.

**TMT-labeled quantitative proteomics**

Jejunal mucosa samples were ground into powder in liquid nitrogen. To each sample, 10 % trichloroacetic acid/acetone was added, followed by washing three times with pre-cooled acetone. The samples were then reconstituted with 8 M urea or subjection to SDS-phenol extraction. Protein concentration was determined using the BCA assay kit (Beyotime) according to the manufacturer's protocol. After overnight trypsin digestion, the peptides were desalted using Strata X C18 (Phenomenex, Torrance, CA, USA) and then freeze-dried under vacuum. The peptides were dissolved in 0.5 M tetraethylammonium bromide (TEAB) and labeled following the instructions of the TMT kit (Thermo Fisher). Labeled peptides were fractionated by high-pH reversed-phase liquid chromatography (RPLC) using an Agilent 300Extend C18 column (5 μm, 4.6 × 250 mm). The fractionated peptides were analyzed by nano LC-MS/MS on the Orbitrap Exploris™ 480 mass spectrometer. After data acquisition, the raw data were processed using Proteome Discoverer (v2.4.1.15) for database searches. Gene Ontology (GO) annotation for the proteome was obtained from the UniProt-GOA database (http://www.ebi.ac.uk/GOA/). Identified proteins were mapped to UniProt IDs and subsequently assigned to GO terms. For proteins lacking GO annotation in the UniProt-GOA database, the InterProScan software was used to predict their GO function based on the protein sequence alignment.

**Microbial 16S rRNA gene sequencing**

Total bacterial DNA was extracted from jejunal mucosa and digesta using the EZNATM Soil DNA kit (D5625-02, Omega Bio-Tek Inc., Norcross, GA, USA) according to the manufacturer's instructions. The V3-V4 hypervariable regions of bacterial 16S rDNA were amplified using a two-step PCR method with primers 338F and 806R, each containing unique 8-bp barcodes for multiplexing. Sequencing was performed on an Illumina sequencing platform using the MiSeq PE300 sequencing protocol. The raw sequencing data were processed using the Majorbio I-Sanger Cloud Platform (www.i-sanger.com), where redundant sequences were removed. Operational taxonomic units (OTUs) were clustered at a 97% similarity cutoff using UPARSE (version 7.1, http://drive5.com/uparse/). Each representative OTU was mapped to the Silva 138 database using the RDP classifier (http://rdp.cme.msu.edu/) at a confidence threshold of 0.7. Further details of the microbial sequencing process are provided in [3].

**Untargeted metabolome**

Jejunal chyme samples were placed in a 2 mL centrifuge tube along with grinding beads. Extraction solvent (methanol: water = 4:1) containing the internal standard L-2-chlorophenylalanine was added for metabolite extraction. The samples were homogenized using a cryogenic grinder, followed by low-temperature ultrasonic extraction. After standing and centrifugation, the supernatant was collected for analysis. Quality control (QC) samples were prepared by pooling metabolites from all samples and inserted during the analysis to monitor reproducibility. The samples were analyzed using ultra-high performance liquid chromatography coupled with tandem mass spectrometry (UHPLC-Q Exactive HF-X). The raw data were imported into Progenesis QI software for processing, and metabolite identification was achieved by matching with metabolic databases (HMDB, Metlin, and an in-house database).

**Statistical analyses**

Microbiome analyses: The bacterial α-diversity indices, including Sobs and Chao index, as well as Principal Co-ordinates Analysis (PCoA) based on Bray-Curtis distance, were calculated to assess microbial diversity using the Majorbio I-Sanger Cloud Platform (www.i-sanger.com). Statistical differences in microbial composition at the genus level and the abundance of *Lactobacillus* species were evaluated via the Wilcoxon-Wallis test for two groups and the Kruskal-Wallis test for comparisons across four groups. A *p*-value of < 0.05 was considered statistically significant, while 0.05 < *p* < 0.10 was regarded as a significant trend.

Proteomics analyses: Protein expression between the CON and XOS groups was compared using a *t*-test. Differentially accumulated proteins (DAPs) were identified based on the *p*-value < 0.1 and a fold-change ≥ 1.20 or ≤ 0.833. Fisher's exact test was then applied to assess the DAPs within the context of the identified proteins, followed by GO or Kyoto Encyclopedia of Genes and Genomes (KEGG) enrichment analyses. A *p*-value (adjusted-*p*-value) < 0.05 was considered statistically significant. Additional functional annotation clustering was performed using DAVID with a significance threshold of *p*-value < 0.05 and Enrichment score > 1.

Metabolomics analyses: Partial Least Squares Discriminant Analysis (PLS-DA) was performed on the preprocessed data matrix using the R package ropls (Version 1.6.2). A 7-fold cross-validation procedure was employed to assess the stability of the models. The selection of significantly different metabolites was based on variable importance in projection (VIP) values obtained from the PLS-DA model and Student’s *t*-test *p*-values. Metabolites with VIP_PLS-DA > 1.49, *p*-value < 0.05 and fold change ≥ 1.2 or ≤ 0.8 were considered significantly different.

General statistical analyses: Data obtained from the expression of specific genes or proteins were analyzed using Student's *t*-test with JMP software (10.0.0 version, SAS Institute, Cary, NC, USA). The two-tailed Student's *t*-test and one way ANOVA were used for two groups and three groups statistical analysis of bile acid data; After a significant ANOVA result, post hoc comparisons were performed using Duncan's multiple range test. The *p* < 0.05 representing statistical significance and 0.05 < *p* < 0.10 representing significant trend. Unless otherwise specified, all data were presented as mean ± SD.

DIABLO analyses: Data Integration Analysis for Biomarker discovery using a Latent component method for Omics (DIABLO) was performed using the “mixOmics” package in the R program environment. The specific details and operations are carried out according to the reference articles from [4] and [5]. The objective is to employ DIABLO for identifying key cross-omics biomarkers via a latent variable model. First, samples were aligned and multi-omics data (including mucosal proteomics, chyme metabolomics, and microbial profiles from both jejunal mucosa and lumen) were standardized, followed by filtering low-variance features. Subsequently, leveraging an extended sparse Generalized Canonical Correlation Analysis (sGCCA) framework, phenotypic labels were incorporated to guide latent component extraction. Key parameters (e.g., number of components, feature count per omics layer) were optimized via cross-validation to maximize both inter-omics correlations and phenotypic discriminative power. Stable non-zero coefficient features were then extracted to construct a cross-omics association network, elucidating interactions among molecular layers. Finally, underlying driver molecules were mined, with key metabolites prioritized as potential biomarkers through integrative analysis.

**REFERENCES**

1. Shatos, Marie A., José D. Ríos, Yoshitaka Horikawa, Robin R. Hodges, Eli L. Chang, Carlo R. Bernardino, Peter A. D. Rubin, Darlene A. Dartt. 2003. “Isolation and characterization of cultured human conjunctival goblet cells.” *Investigative Ophthalmology & Visual Science* 44: 2477-2486. https://doi.org/10.1167/iovs.02-0550

2. Tang, Shanlong, Jingjing Xie, Wei Fang, Xiaobin Wen, Chang Yin, Qingshi Meng, Ruqing Zhong, Liang Chen, Hongfu Zhang. 2022. “Chronic heat stress induces the disorder of gut transport and immune function associated with endoplasmic reticulum stress in growing pigs.” *Animal Nutrition* 11: 228-241. https://doi.org/10.1016/j.aninu.2022.08.008

3. Tang, Shanlong, Ruqing Zhong, Chang Yin, Dan Su, Jingjing Xie, Liang Chen, Lei Liu, Hongfu Zhang. 2021. “Exposure to High Aerial Ammonia Causes Hindgut Dysbiotic Microbiota and Alterations of Microbiota-Derived Metabolites in Growing Pigs.” *Frontiers in Nutrition* 8: 689818. https://doi.org/10.3389/fnut.2021.689818

4. Singh, Amrit, Casey P. Shannon, Benoît Gautier, Florian Rohart, Michaël Vacher, Scott J. Tebbutt, Kim-Anh Lê Cao. 2019. “DIABLO: an integrative approach for identifying key molecular drivers from multi-omics assays.” *Bioinformatics* 35: 3055-3062. https://doi.org/10.1093/bioinformatics/bty1054

5. Tang, Shanlong, Yuxia Chen, Fuli Deng, Xiaowei Yan, Ruqing Zhong, Qingshi Meng, Lei Liu, et al. 2022. “Xylooligosaccharide-mediated gut microbiota enhances gut barrier and modulates gut immunity associated with alterations of biological processes in a pig model.” *Carbohydrate Polymers* 294: 119776. https://doi.org/10.1016/j.carbpol.2022.119776

**
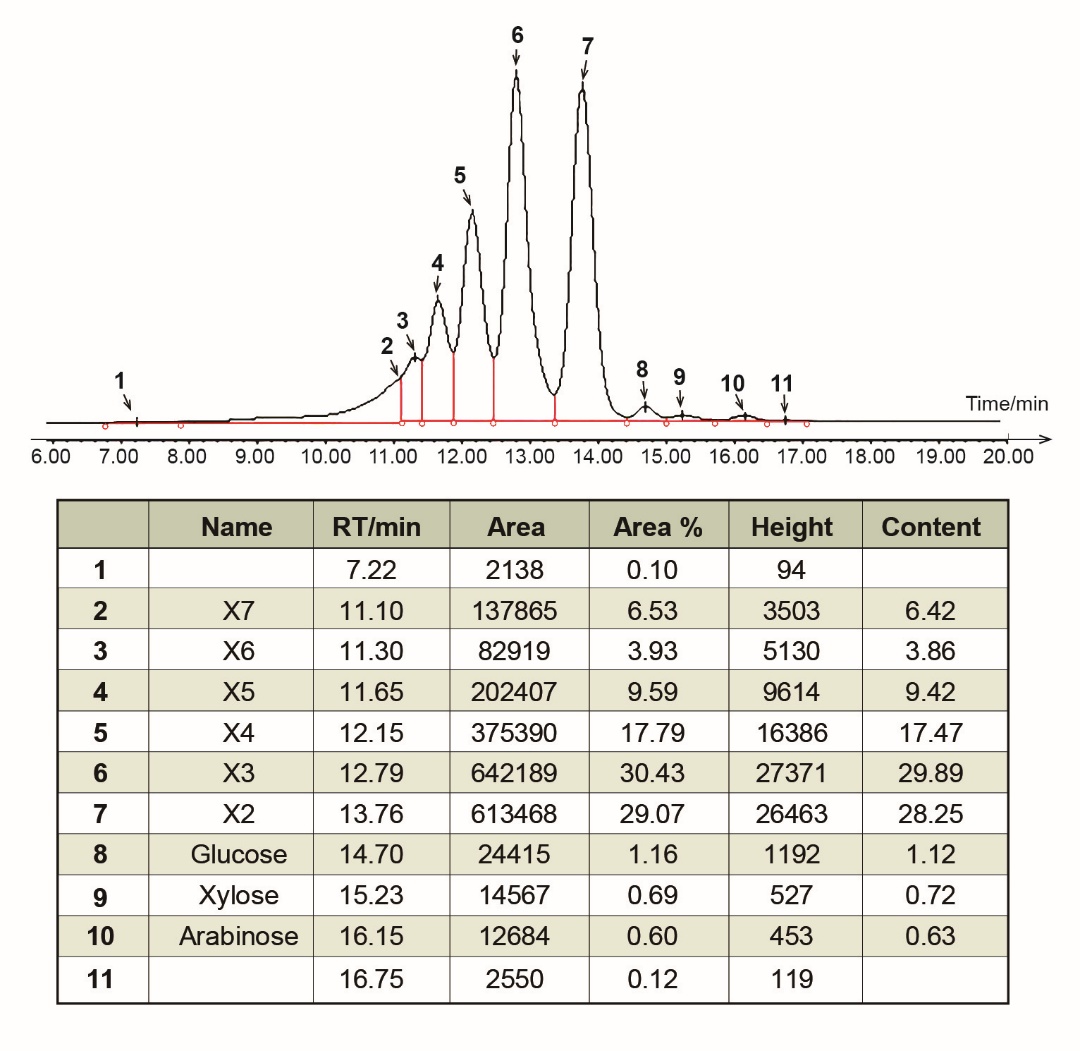
**

**Figure S1 The components of XOS and their corresponding retention times.** X2, Xylobiose; X3, Xylotriose; X4, Xylotetraose; X5, Xylopentaose; X6, Xylohexaose; X7, Xyloheptaose; RT, Retention time.

**
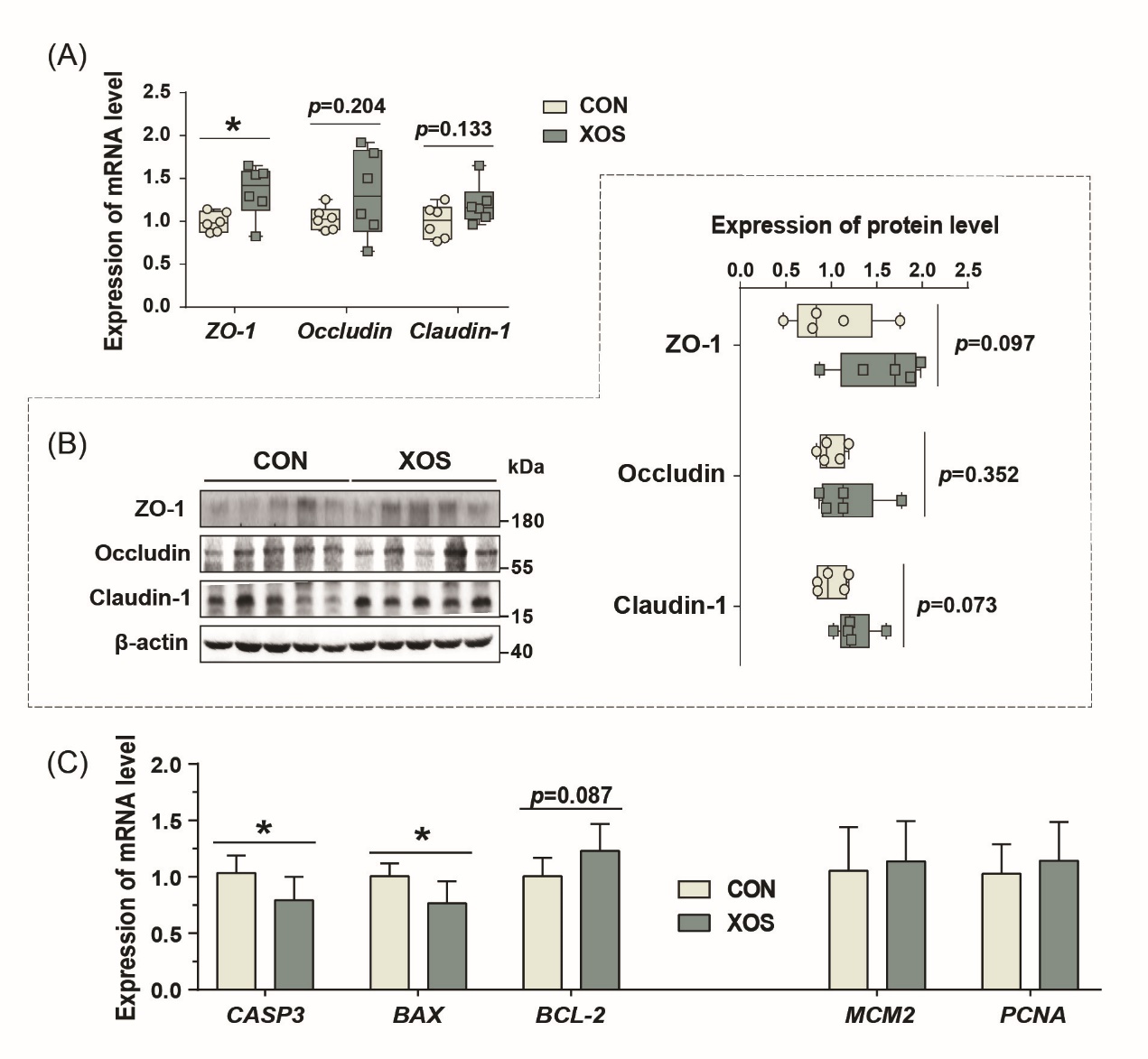
Figure S2 Gene expression related to intestinal tight junction, cell apoptosis or proliferation after XOS supplement.** The mRNA (A) or protein level (B) of tight junction proteins in the jejunum. The mRNA (C) of cell apoptosis- and proliferation-related genes in the jejunal mucosa of piglets after XOS addition. Values are mean ± SD or mix to max with all points shown (n = 5−6), a two-tailed Student's *t*-test was used for statistical analysis with asterisks denoting significant differences (**p* < 0.05). The CON group represents pigs fed a basal diet, while the XOS group includes pigs fed a basal diet supplemented with 500 mg/kg XOS.

**
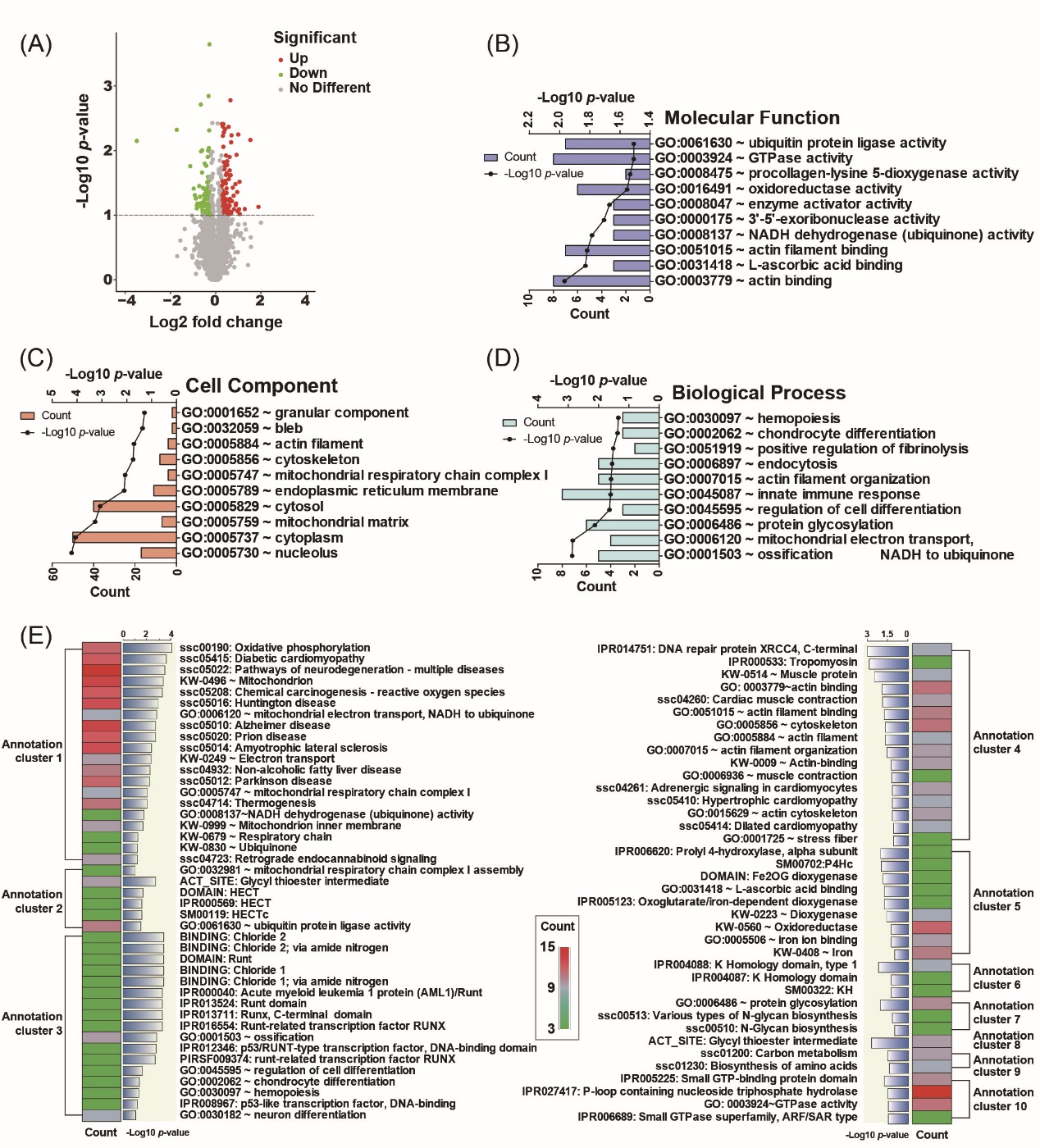
Figure S3 Enrichment analysis of RNA sequencing.** (A) Volcano plot for whole identified proteins in CON vs. XOS groups. Gene Ontology (GO) annotation of enriched pathways of differentially expressed genes, including Molecular Function (MF, B), Cell Component (CC, C) and Biological Process (BP, D). (E) Functional annotation clustering of differentially accumulated proteins (DAPs) via DAVID online analysis.

**
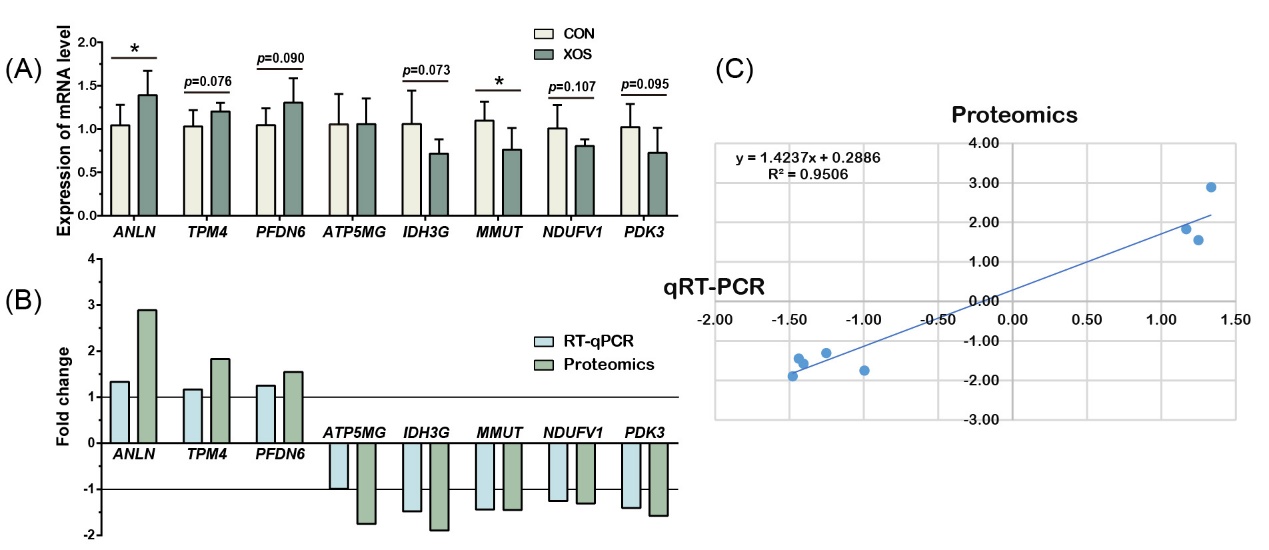
Figure S4 Validation of proteomics analysis results.** (A) Validation of eight genes associated with identified DAPs by qRT-PCR. Fold change (B) and correlation analysis (C) of selected proteins between qRT-PCR validation and proteomics analysis. Data are presented as mean ± SD (n = 6). A two-tailed Student’s *t*-test was used for statistical comparisons with * indicating significant differences (**p* < 0.05).

**
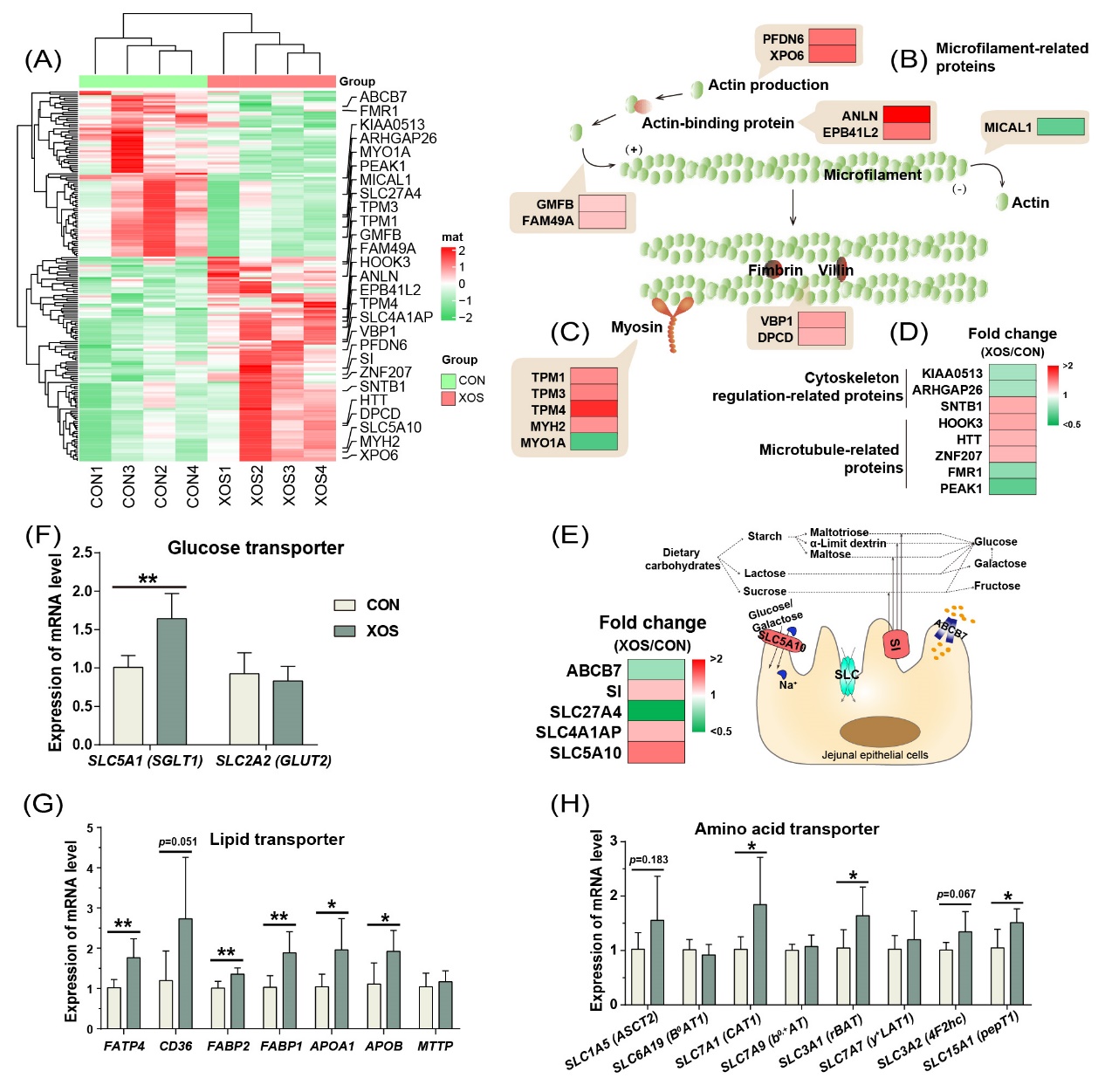
Figure S5** **The expression of nutrient transporter genes and cytoskeleton-related proteins from proteomic or qRT-PCR data.** (A) Heat map of hierarchical cluster analysis showing differentially accumulated proteins related to nutrient transport and the cytoskeleton. Effects of XOS supplementation on proteins associated with cytoskeleton (B−D) and nutrient transport (E). The mRNA expression levels of glucose (F), lipid (G) and amino acid (H) transporters after XOS supplementation. Values are mean ± SD, n = 4 for proteomic data and n = 6 for gene expression. A two-tailed Student's *t*-test was used for statistical analysis with asterisks denoting significant differences (**p* < 0.05 and ***p* < 0.01).

**
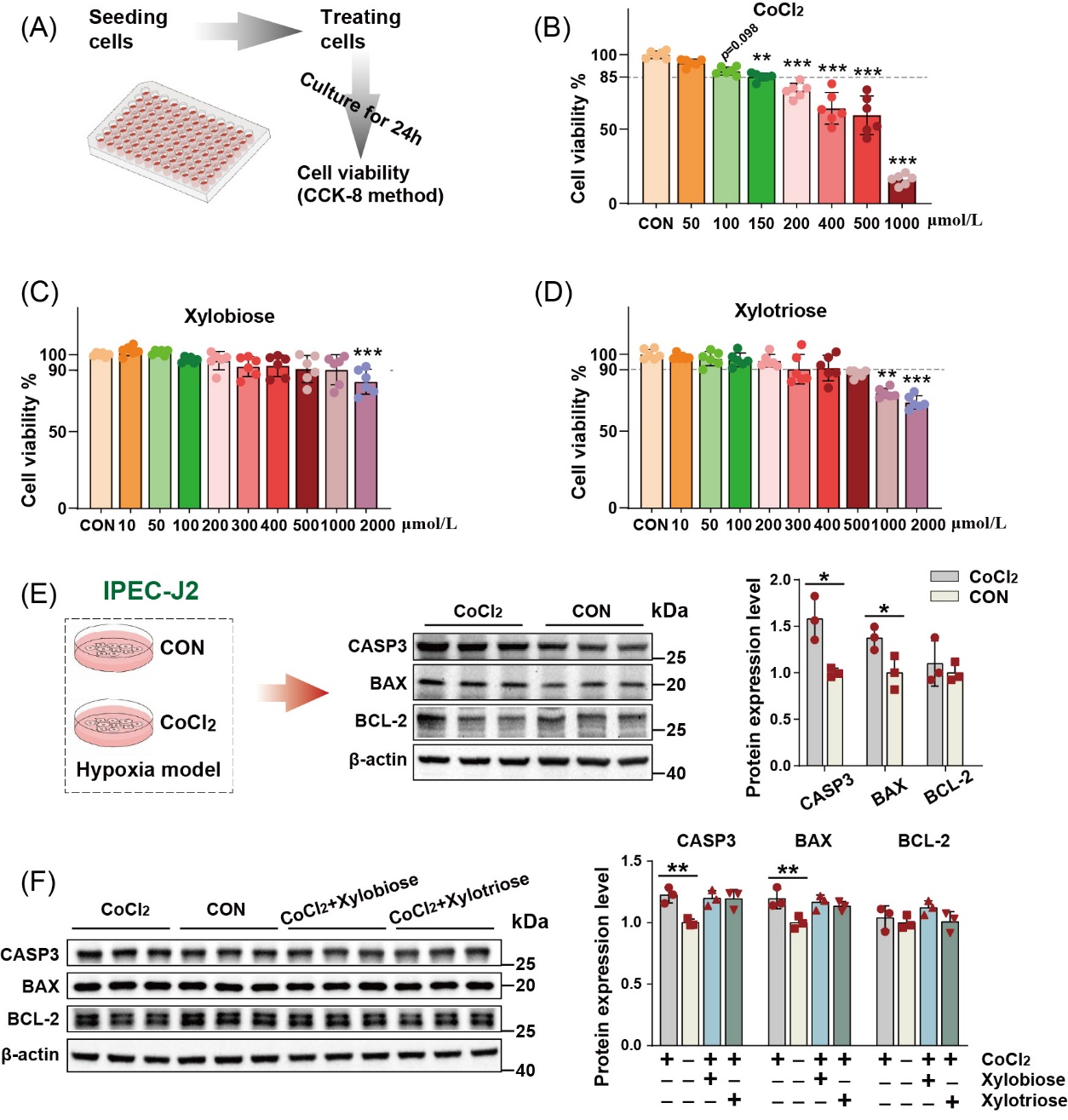
**

**Figure S6 The hypoxia-induced apoptosis model and effects of xylobiose or xylotriose on attenuating hypoxia-induced apoptosis of IPEC-J2 cells *in vitro*.** (A) Process of cytotoxicity experiment of additives; Cytotoxicity experiment of exogenous CoCl_2_ (B), xylobiose (C) and xylotriose (D) through the CCK-8 method. (E) Relative expression of apoptosis-related proteins in IPEC-J2 cells after CoCl_2_ supplementation-induced hypoxia model; (F) Relative expression of apoptosis-related proteins in hypoxia-model cells with xylobiose/xylotriose supplementation. Values are expressed as mean ± SD (n = 3 or 6). A two-tailed Student's *t*-test was used for statistical analysis and asterisks denote significant differences (**p* < 0.05, ***p* < 0.01 and ****p* < 0.001). The treatment concentration of CoCl_2_ and xylobiose/xylotriose is 300 μM and 400 μM.

**
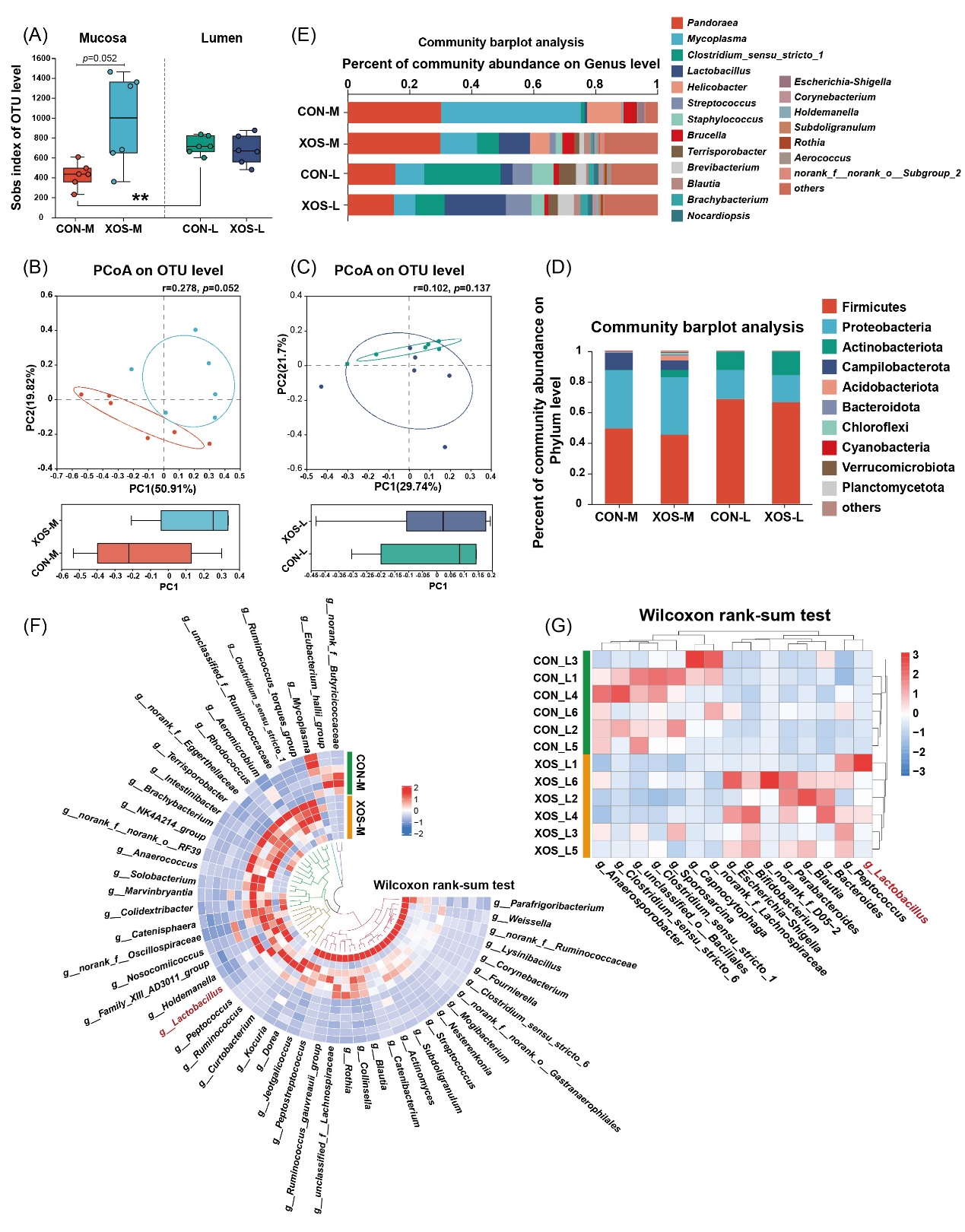
**

**Figure S7 The changes of microbial composition.** (A) Sobs index at OTU level. (B−C) PCoA analysis of the jejunal luminal and mucosal microbiome based on Bray-Curtis distance metrics at OTU level. Community barplot analysis at phylum level (D) or genus level (E). Nonparametric tests for mucosal (CON-M vs. XOS-M, F) and luminal (CON-L vs. XOS-L, G) microbiota abundance at Genus level, as well as α-diversity analysis (n = 6). Asterisk denotes significant differences (**p* < 0.05 and ***p* < 0.01).

**
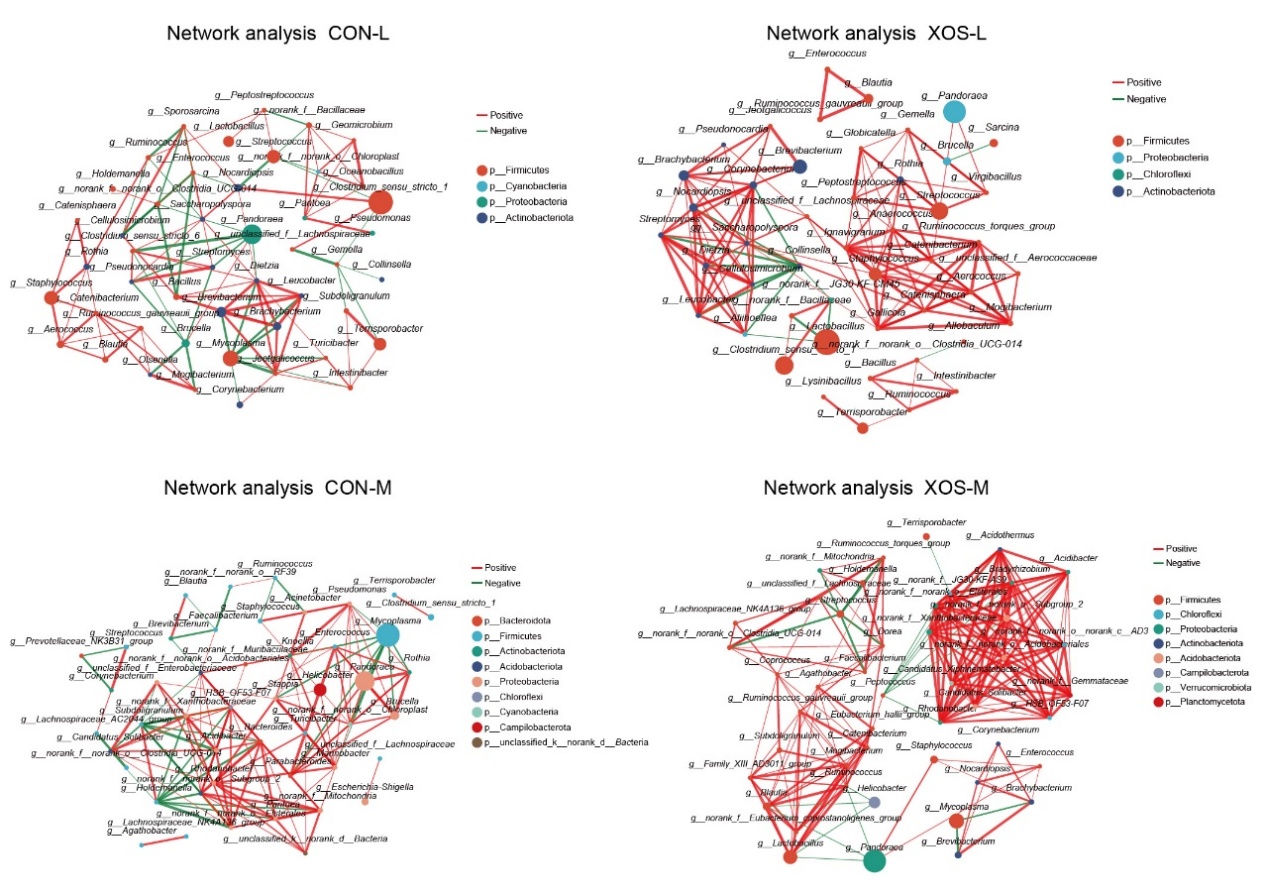
**

**Figure S8 A network for correlation analysis in the relative abundances of microbiota genera.** Only correlations with a Spearman’s coefficient > 0.7 and a *p* < 0.05 were shown. The different color of nodes indicates various microbiota phyla as described in legend, and the size of nodes indicates species abundance. The thickness of the line represents the size of the correlation coefficient. The line color represents correlation, with red representing positive correlation and green representing negative correlation. CON: control group pigs fed a basal diet, XOS: XOS group pigs fed a basal diet supplemented with 500 mg/kg XOS; L means jejunal lumen and M means jejunal mucosa.

**
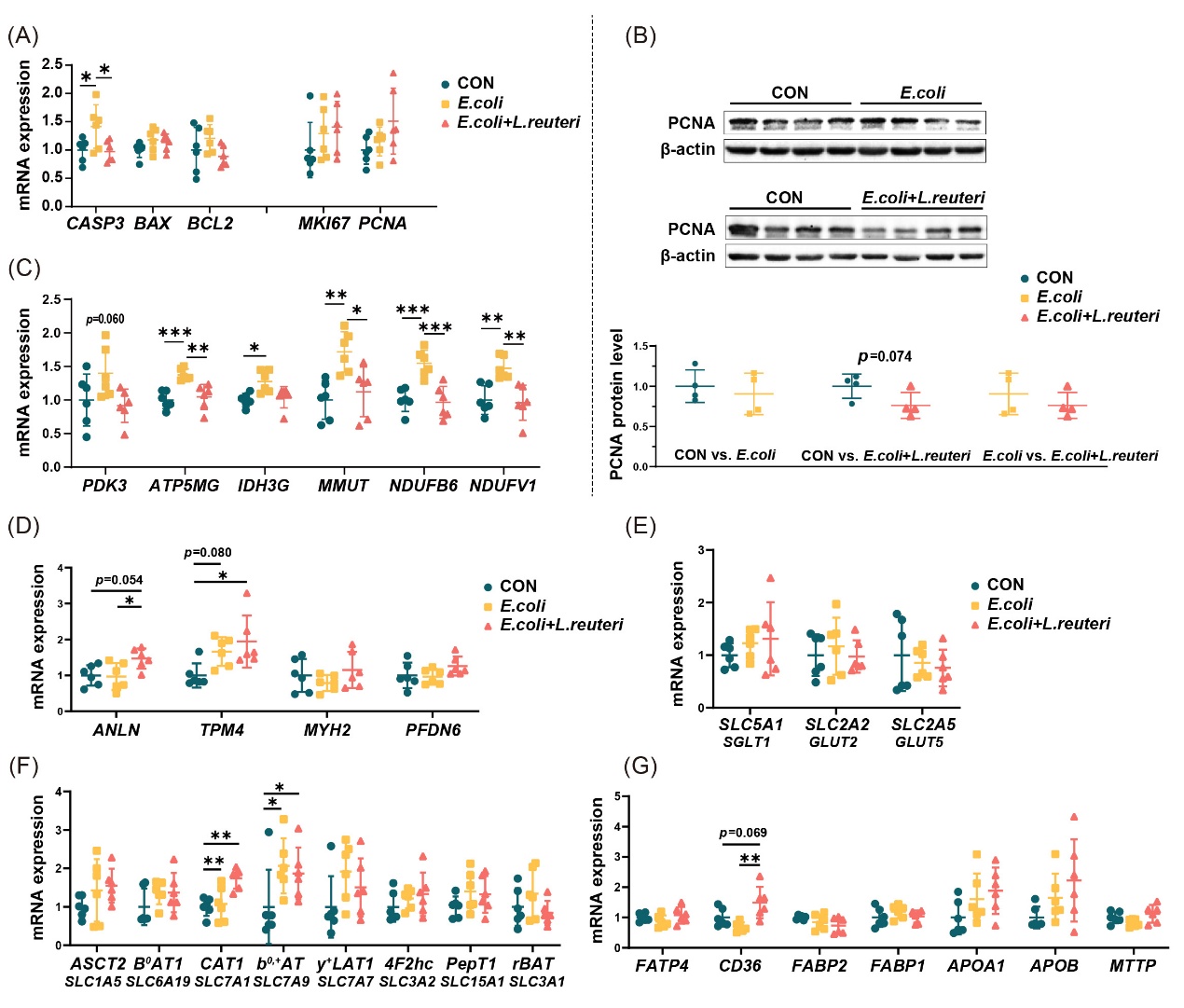
**

**Figure S9 Protein and gene expression in jejunal mucosa after *E. coli* injection with or without *L. reuteri* supplement.** The genes expression related to cell apoptosis and proliferation (A), energy metabolism (C), cytoskeleton (D), glucose (E), amino acid (F) and fatty acid (G) transporters. (B) The protein expression of PCNA in jejunal mucosa. Values are mean ± SD. A two-tailed Student's *t*-test was used for statistical analysis and asterisks denote significant differences (**p* < 0.05, ***p* < 0.01 and ****p* < 0.001). The CON group: piglets receiving a basal diet, the *E. coli* group: piglets receiving a basal diet with several *E. coli* injection, and *E. coli* + *L. reuteri* group: piglets receiving a basal diet containing 1.0 × 10^8^ CFU *Lactobacillus reuteri* with several *E. coli* injection.

**
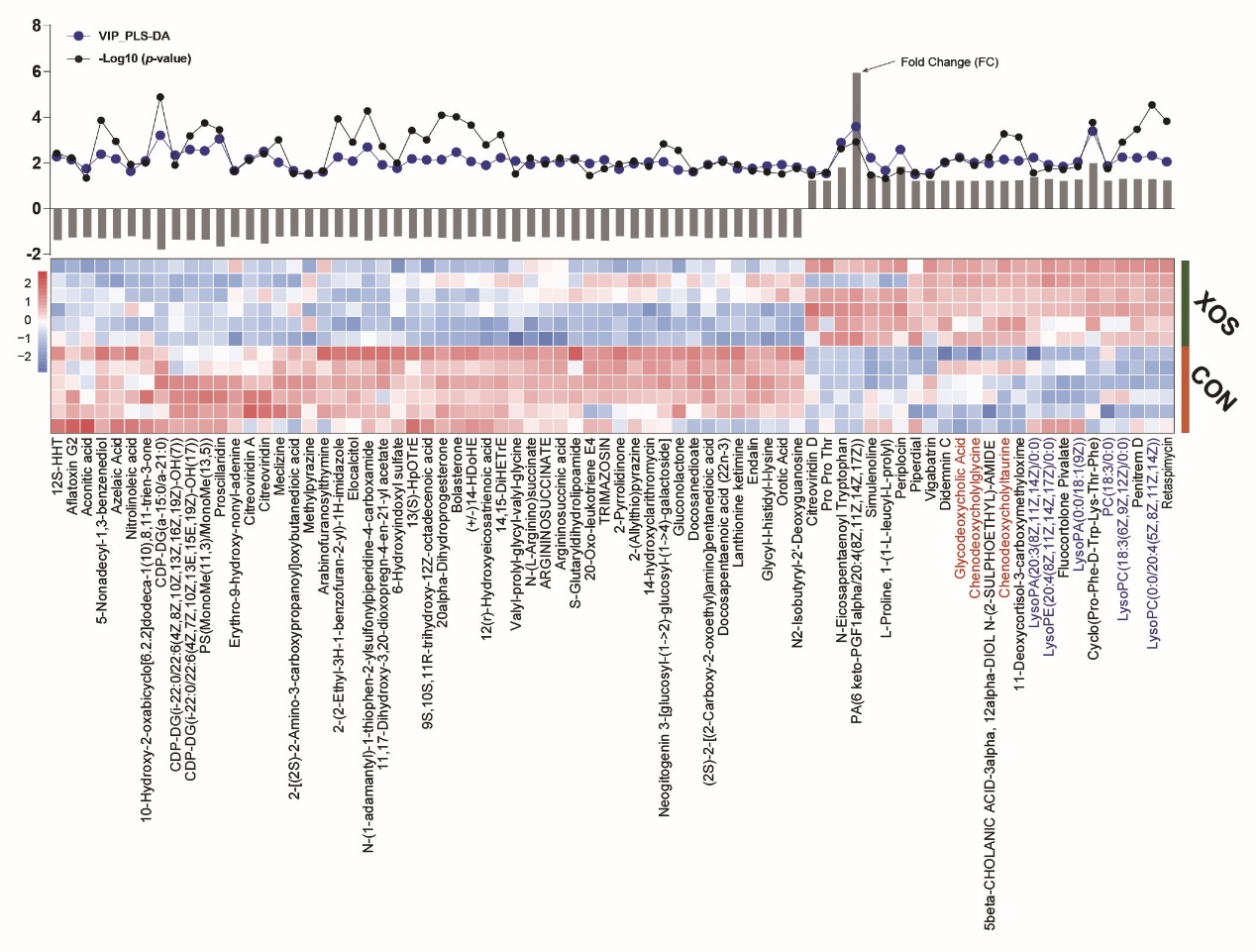
Figure S10 Differential metabolite profile for jejunal digesta (CON vs XOS).**

**
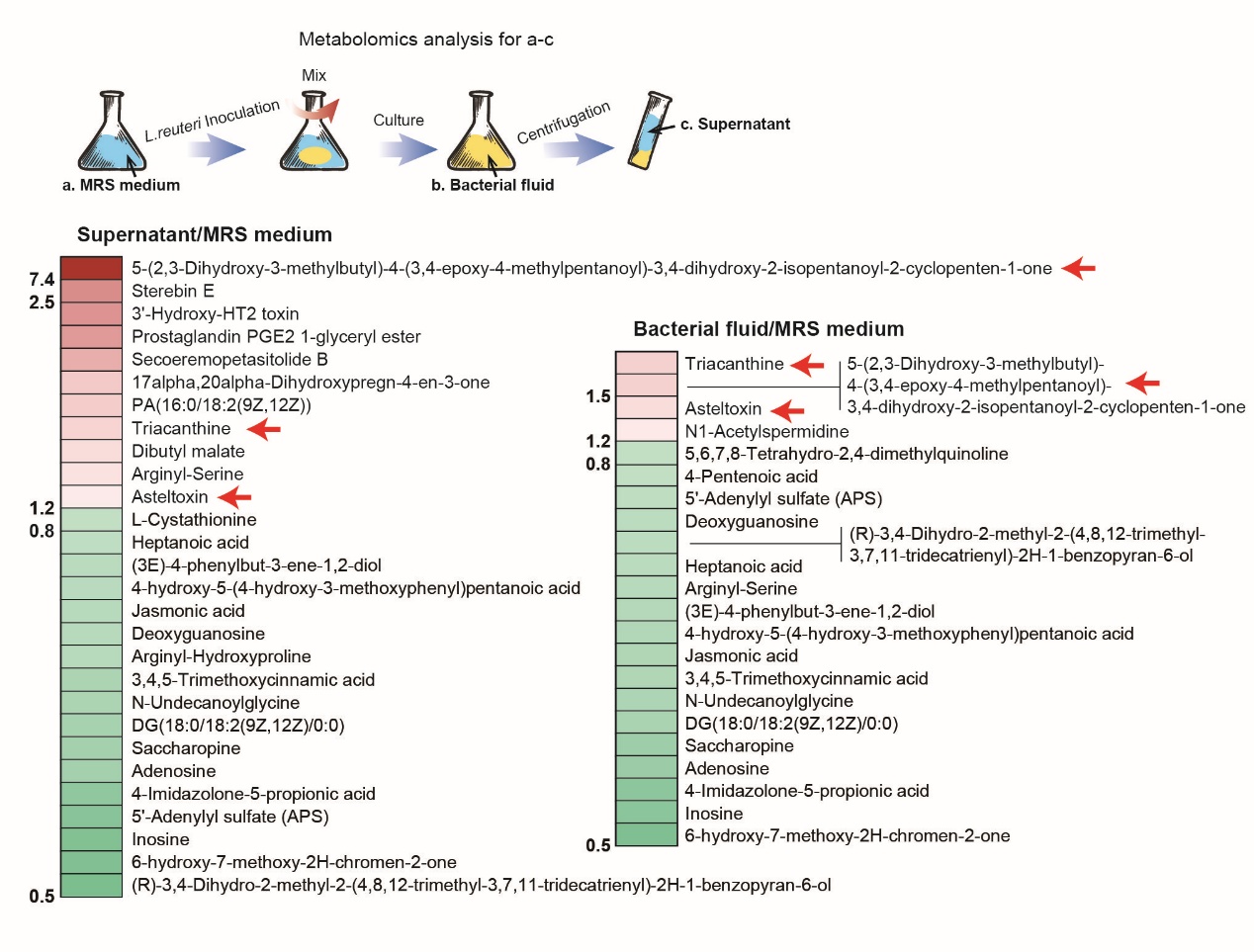
Figure S11 Metabolomics of bacteria.** The non-target metabolomics of bacterial suspension (b. bacterial fluid in figure) and culture supernatants (c. supernatant in figure) detected potential metabolites which may be produced by *L. reuteri*.

**
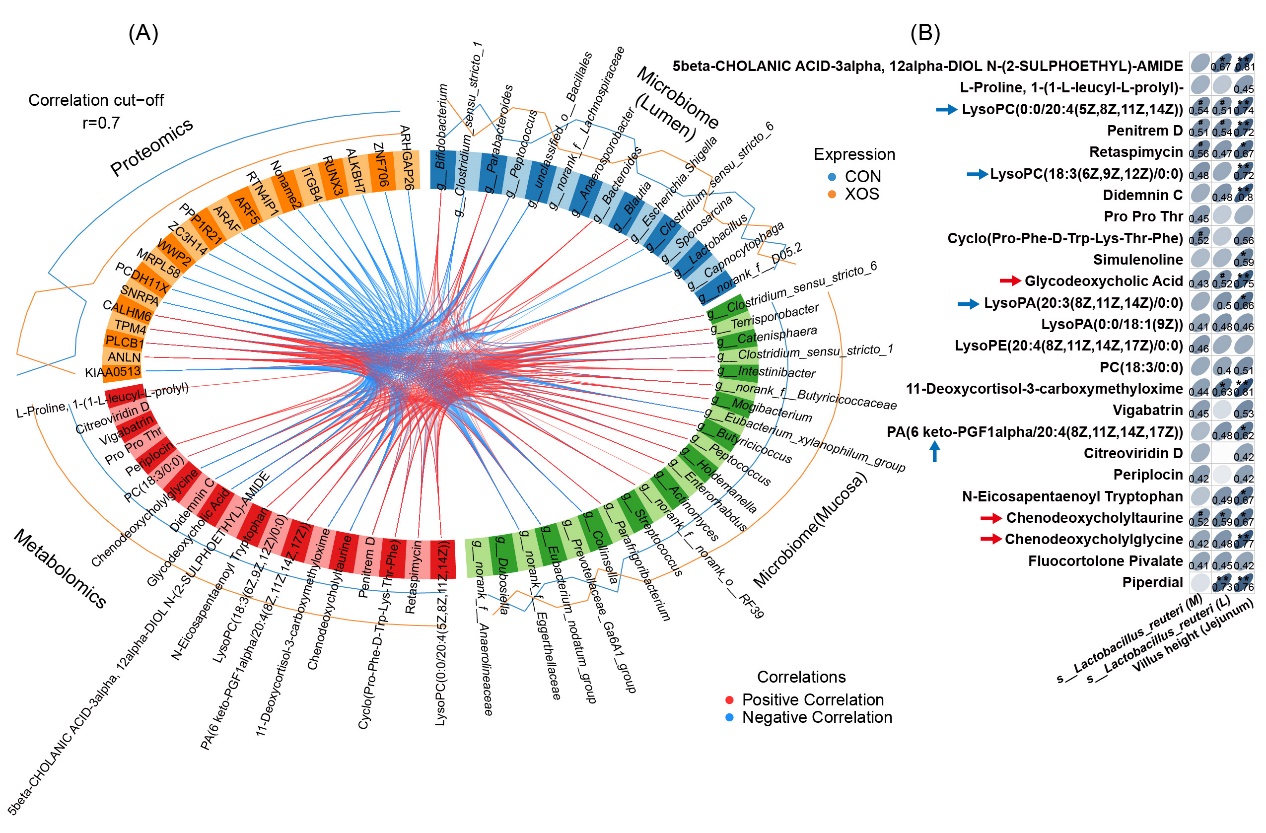
Figure S12 Metabolomic data and multi-omics correlation analysis.** Correlation analysis for proteome, microbiome (jejunal lumen and mucosa) and metabolome (jejunal digesta) by DIABLO (Data Integration Analysis for Biomarker discovery using a Latent component method for Omics). (A) The circos plot displays correlations among different omics components (metabolomic data only uses up-regulated metabolites). (B) Pearson correlation between up-regulated metabolites in XOS group and gut index or microbiota including *L. reuteri* in jejunal lumen or mucosa (# *p* < 0.1, * *p* < 0.05, ** *p* < 0.01).

**
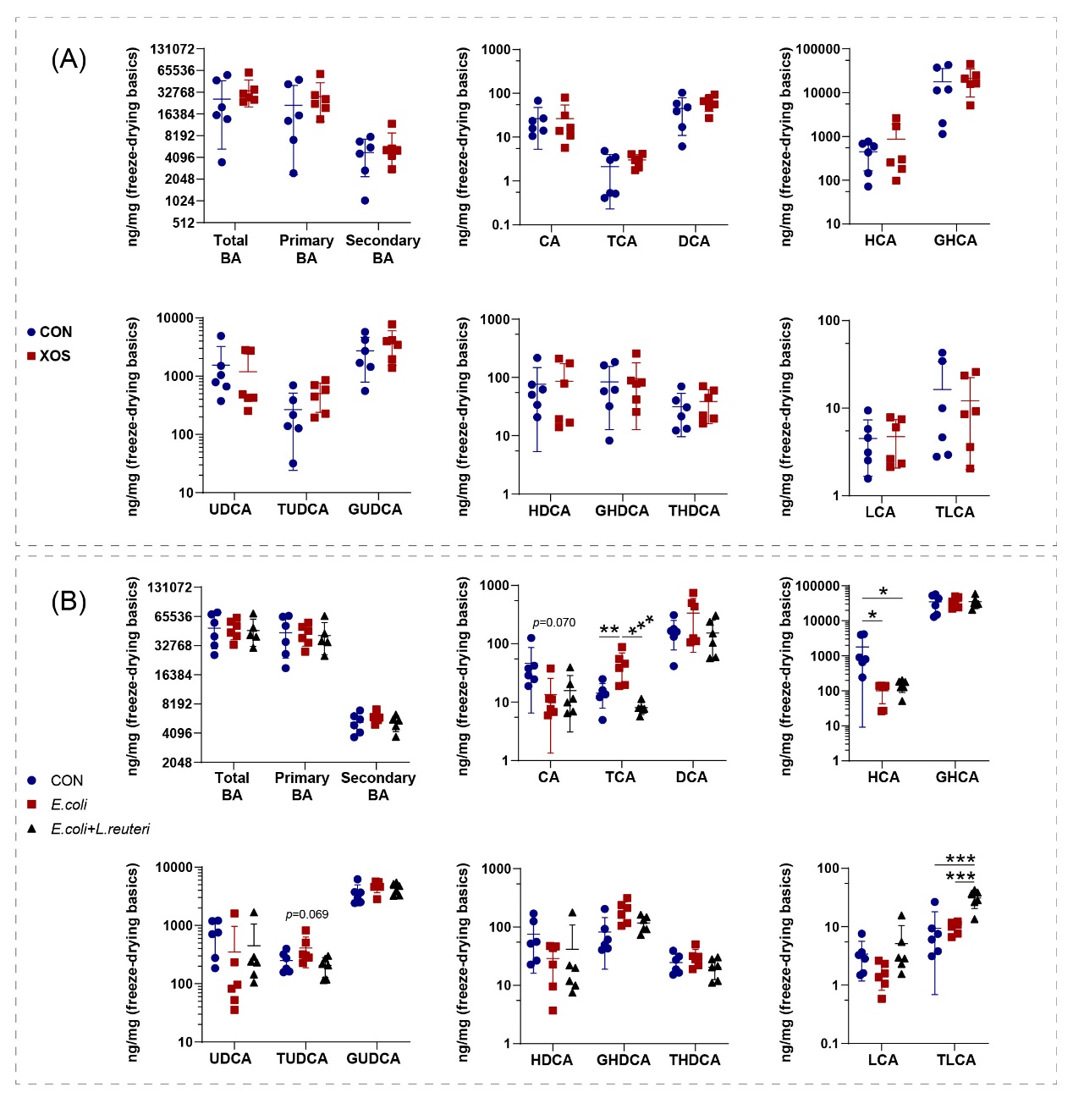
Figure S13 Bile acid contents.** The bile acid contents of jejunal digesta in XOS-treated group pigs (A) or the *E. coli + L. reuteri* group pigs (B) through targeted metabolome analysis. Values are mean ± SD (n = 6) and a two-tailed Student's *t*-test was used for statistical analysis. The *, ** and *** represent *p* < 0.05, *p* < 0.01 and *p* < 0.001, respectively.

**
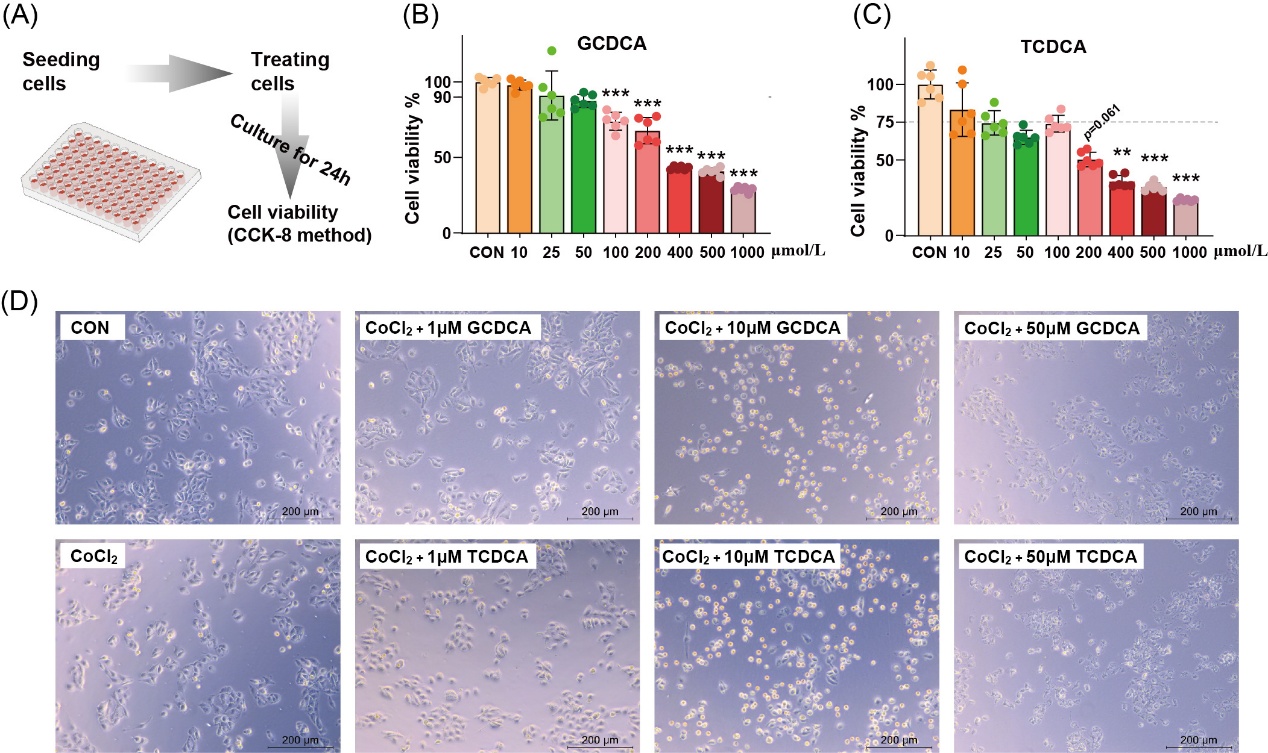
**

**Figure S14 Cytotoxicity and tolerance experiments of exogenous additives.** (A) Process of cytotoxicity experiment of additives; Cytotoxicity experiment of exogenous GCDCA (B), TCDCA (C) through the CCK-8 method; (D) Alterations in cell morphology after adding different concentrations of additives to CoCl_2_-induced hypoxia in IPEC-J2 cells. Values are mean ± SD (n = 6). A two-tailed Student's *t*-test was used for statistical analysis and asterisks denote significant differences (***p* < 0.01 and ****p* < 0.001).
